# Dendritic cell targeted HIV‐1 gag protein vaccine provides help to a recombinant Newcastle disease virus vectored vaccine including mobilization of protective CD8^+^ T cells

**DOI:** 10.1002/iid3.209

**Published:** 2017-12-04

**Authors:** Loveline N. Ngu, Nadesh N. Nji, Georgia Ambada, Apeh A. Ngoh, Ghislain D. Njambe Priso, Jules C. Tchadji, Abel Lissom, Suzanne H. Magagoum, Carol N. Sake, Thibau F. Tchouangueu, George O. Chukwuma, Arinze S. Okoli, Bertrand Sagnia, Rebecca Chukwuanukwu, Denis M. Tebit, Charles O. Esimone, Alain B. Waffo, Chae G. Park, Klaus Überla, Godwin W. Nchinda

**Affiliations:** ^1^ Department of Biochemistry University of Yaounde One P.O. Box 812 Yaounde Cameroon; ^2^ Laboratory of Vaccinology/Biobanking of The Chantal Biya International Reference Center for research on the prevention and management of HIV/AIDS (CIRCB) BP 3077 Messa Yaounde Cameroon; ^3^ Microbiology and Immunology Laboratory CIRCB Yaounde Cameroon; ^4^ Department of Animal Biology and Physiology University of Yaounde One P.O. Box 812 Yaounde Cameroon; ^5^ Department of biomedical sciences University of Dschang Dschang Cameroon; ^6^ Department of Microbiology University of Yaounde One P.O. Box 812 Yaounde Cameroon; ^7^ Department of biochemistry University of Dschang Dschang Cameroon; ^8^ Department of Medical Laboratory Science College of Medicine, Nnewi Campus Nnamdi Azikiwe University Awka Anambra; ^9^ GenØk − Centre for Biosafety Tromsø Norway; ^10^ Myles Thaler Center for AIDS and Human Retrovirus Research Department of Microbiology, Immunology and Cancer Biology Jordan Hall 7088, 1340 Jefferson Park Avenue Charlottesville Virginia 22903 USA; ^11^ Department of Pharmaceutical Microbiology and Biotechnology Nnamdi Azikiwe University Awka Nigeria; ^12^ Department of Biological Sciences # 223 Alabama State University 1627, Hall Street Montgomery Alabama 36104 USA; ^13^ Laboratory of Immunology, Brain Korea 21 PLUS Project for Medical Science, Severance Biomedical Science Institute Yonsei University College of Medicine Seoul 03722 Republic of Korea; ^14^ Laboratory of Cellular Physiology and Immunology and Chris Browne Center for Immunology and Immune Diseases Rockefeller University New York New York 10065 USA; ^15^ Institute of Clinical and Molecular Virology University Hospital Erlangen Erlangen Germany

**Keywords:** Complementary prime boost, immunity, murine airway, polyfunctional T cells, protective

## Abstract

**Introduction:**

Recombinant Newcastle Disease virus (rNDV) vectored vaccines are safe mucosal applicable vaccines with intrinsic immune‐modulatory properties for the induction of efficient immunity. Like all viral vectored vaccines repeated inoculation via mucosal routes invariably results to immunity against viral vaccine vectors. To obviate immunity against viral vaccine vectors and improve the ability of rNDV vectored vaccines in inducing T cell immunity in murine air way we have directed dendritic cell targeted HIV‐1 gag protein (DEC‐Gag) vaccine; for the induction of helper CD4^+^ T cells to a Recombinant Newcastle disease virus expressing codon optimized HIV‐1 Gag P55 (rNDV‐L‐Gag) vaccine.

**Methods:**

We do so through successive administration of anti‐DEC205‐gagP24 protein plus polyICLC (DEC‐Gag) vaccine and rNDV‐L‐Gag. First strong gag specific helper CD4^+^ T cells are induced in mice by selected targeting of anti‐DEC205‐gagP24 protein vaccine to dendritic cells (DC) *in situ* together with polyICLC as adjuvant. This targeting helped T cell immunity develop to a subsequent rNDV‐L‐Gag vaccine and improved both systemic and mucosal gag specific immunity.

**Results:**

This sequential DEC‐Gag vaccine prime followed by an rNDV‐L‐gag boost results to improved viral vectored immunization in murine airway, including mobilization of protective CD8^+^ T cells to a pathogenic virus infection site.

**Conclusion:**

Thus, complementary prime boost vaccination, in which prime and boost favor distinct types of T cell immunity, improves viral vectored immunization, including mobilization of protective CD8^+^T cells to a pathogenic virus infection site such as the murine airway.

## Introduction

The high genetic variability of the human immunodeficiency virus type 1 (HIV‐1) and the tendency to persist in hidden viral reservoir have seriously hampered the development of safe and effective vaccines (reviewed in Ref. 1, 2, 3, 4, 5). More so Lifelong highly active anti‐retroviral therapy (HAART) is limited by the continuous emergence of drug resistance and is not curative (reviewed in Ref. 6). Since they can selectively kill virus producing cells the induction of potent HIV‐1 specific T cell immunity as a component of prophylactic or therapeutic vaccine remains a highly desired objective (7, 8 reviewed in Ref. 1). The presence of robust multifunctional T cell immunity for example has been associated with long term viremia suppression of HIV‐1 in Elite controller (9, 10, rev. in Ref. 11) and has also been implicated in preclinical studies in the control of pathogenic Simian immunodeficiency virus infections 12, 13.

Viral vectors are the most efficient vaccine vectors and are being actively optimized for immunization against infectious diseases and cancers (rev. in Ref. 14). Unfortunately several clinical evaluation of viral vectored HIV‐1 vaccines in multiple heterologous prime boost combinations have yielded limited success 15, 16, 17, 18. In addition to the poor immunogenicity of some of these vaccine candidates a major obstacle has been pre‐existing or the risk of the induction of anti‐vector immunity during the course of immunization 19, 20. We previously reported that preprograming of helper CD4^+^ T cells using dendritic cells targeted protein‐based vaccine enables DNA vaccines to be more immunogenic in mice 22. One component of the improved protection was a more rapid accumulation of gag‐specific CD8^+^ T cells to a mucosal infection challenge site. Previously to improve viral vectored HIV‐1 gag vaccine we targeted the encoded antigen to dendritic cells in situ using either adenoviral or rNDV vectored HIV‐1 vaccine leading in enhanced T cell immunity 21. In this study we have demonstrated that successive administration of anti‐DEC205‐gagP24 protein plus polyICLC and rNDV‐L‐gag vaccines results to vigorous systemic and mucosal T cell immunity. Here antigen specific helper CD4^+^ T cells are elicited by a priming dose of a DEC‐targeted protein vaccine which then improves the induction of T cell immunity by a rNDV‐L‐gag vaccine. In addition to an enhanced T cell immunity; this approach strongly amplified persistent CD8^+^ T cells in murine airway and improved protection against airway challenge with a recombinant vaccinia gag virus. To the best of our knowledge, this is the first viral vectored—dendritic cell targeted protein vaccine combination strategy in mice in which the concept of helper CD4^+^ T cells has been exploited not only to direct the induction of a balanced HIV‐1 gag specific T cell immunity but equally to intentionally focus protective CD8^+^ T cell responses at the site of pathogenic virus challenge. In contrast to our previous findings on complementary prime boost vaccination where a more rapid accumulation of gag‐specific CD8^+^ T cells occurred upon pathogenic virus challenge, here enhanced accumulation of vaccine induced T cells to the lungs is driven by mucosal rNDV‐L‐gag application.

We previously used the term “complementary” prime boost to describe a DEC‐gag prime plus DNA boost 22. Here we extend this concept to include DEC‐gag prime plus rNDV‐L‐gag boost where an anti‐DEC205‐gagP24 plus polyICLC protein vaccine is combined with viral vectored vaccine to enhance protective T cell immunity in murine airway.

## Materials and Methods

### Mice and rNDV‐L‐gag vaccination

CxB6 F1 mice from Harlan were maintained under specific‐pathogen free conditions and used at 6–10 weeks under the guidance of the Rockefeller University Institutional Animal Care and Use Committee. Mice were inoculated with rNDV‐L‐gag or rNDV‐L‐GFP intranasally as previously reported 21. The mice were anesthetized with Halothane. On the other hand DEC‐gag plus polyICLC injection was given subcutaneously in the paws as previously reported 22, 23.

### Ethical statement

Mice were maintained under specific‐pathogen free conditions and used at 6–10 weeks under the guidance of the Rockefeller University Institutional Animal Care and Use Committee.

### Cell lines, media, and antibodies

A 293 T (293ts/A1609), CHOneo cells, and CHOmDEC205 cell lines were maintained in DMEM supplemented with 5% FCS, penicillin, streptomycin and glutamine. CV‐1 cells were maintained in this medium but with geneticin in place of streptomycin. Antibodies to CD3, CD4, CD8α, and cytokines (IFN‐γ, IL‐2, and TNF‐α) were purchased from BD Biosciences‐Pharmingen, San Jose, CA. Other antibodies were from Beckman coulter including FITC labeled (KC57‐FITC; Coulter) or HRP‐conjugated antibody (ImmunoDiagnostics, Woburn, MA) to HIV gag p24 and HIV gag CD8^+^ tetramer (AMQMLKETI) H‐2Kd PE.

#### Fusion HIV‐1 gag mAbs

These were generated as described 24, 25. SDS/PAGE was used to assess the quality of the anti‐DEC‐gagP24 (DEC‐gag) fusion proteins, while western blotting using HRP‐anti‐p24 (ImmunoDiagnostics) was used to determine the specificity of the gag fusion constructs. mAb binding was verified on CHO cells stably transfected with the mouse DEC‐205 by FACS, using either PE‐conjugated goat anti‐mouse IgG (Jackson ImmunoResearch, West grove, PA) or FITC‐conjugated anti‐p24 (KC57‐FITC, coulter). All mAbs were endotoxin‐free in limulus Amebocyte Lysate assay, QCL‐1000 (Cambrex, Walkersville, MD).

#### 15‐mer peptide libraries

Overlapping (staggered by 4 aa) 15‐mer peptides spanning the entire HIV‐1 gag p24 25 were synthesized by the proteomic Resource Center (The Rockefeller University). The resuspension had previously been reported in 22, 23, 25.

#### Immunizations

Female CXB6 F1 mice were injected once subcutaneously as described above with 5ug DEC‐gag together with poly‐ICLC (50 μg Oncovir, NW Washington, DC) as adjuvant. 4 wks later the mice received either a boost with 1 × 10^6^ rNDV‐L‐gag i.n. or a second dose of 5ug DEC‐gag plus 50 ug poly‐ICLC subcutaneously Polyinosinic–polycytidylic acid‐polylysine‐carboxymethylcellulose (poly‐ICLC, Hiltonol; Oncovir, Washington, DC, USA) is a stabilized double‐stranded RNA (dsRNA).

#### Assays for HIV‐specific immune T cells

To determine the breadth of HIV‐1 gag‐specific T cell responses, bulk splenocytes were restimulated in vitro either with peptides mix spanning the entire gagP24 protein 24, 25 or a negative unreactive control peptide mix consisting of HIV‐1 nef pool mix in the presence of 2 μg/ml of anti‐CD28 (clone 37.51) for 6 h, adding 10 ug/ml brefeldin A (Sigma–Aldrich, St. Louis, MO) for the last 4 h to accumulate intracellular cytokines. Dead cells were excluded using live/dead fixable dead stain kit (Aqua live/dead; Invitrogen). After blocking Fcγ receptors, the cells were stained with antibodies to CD3‐pacific blue, CD4‐percp, CD8‐alexa‐750, and Aqua live/dead stain for 20 min at 37°C. Cells were washed, fixed (Cytofix/cytoperm; BD Bioscience), permeabilized with Permwash and stained with antibodies to IFNγ (IFNγ‐alexa‐700), IL‐2 (IL‐2‐FITC), and TNF‐α (TNF‐α‐PE‐CY7) for 15 min at room temperature. All antibodies were from Ebioscience. We use BD LSRII for acquisition with data analysis in Flowjo (Tree Star, Inc).

#### HIV‐1 gag CD8^+^ tetramer staining

Lymphocytes were washed once with PBS and stained with HIV‐1 gag CD8 tetramer (AMQMLKETI) H‐2Kd PE, CD3‐pacific blue, CD4‐percp, CD8‐alexa‐700, and Aqua live/dead for 20 min at 4°C as previously described 22, 24.

#### Vaccinia gag protection assays

Nembutal‐anesthetized mice were challenged with 10^5^ PFU/mouse of infectious recombinant vaccinia gag virus by the intranasal route, in 35 μl PBS with Mg/Ca. The negative controls were wild type vaccinia virus, Vaccinia‐luciferase or vaccinia‐OVA virus. The weight of each animal (groups of 5) was determined daily for 7 days following challenge. Then mice were euthanized, the lungs were harvested and homogenized in transport medium (0.1% gelatin in PBS), and stored in duplicates at −80°C prior to virus titration. Lung virus titers of individual mice in each group were determined by plaque assay on monolayers of CV‐1 cells as described 21, 22, 23, 24, 25, 26.

### CD8+ and CD4+ T cell depletion

Vaccinated animals were depleted of CD8+ T‐cells, by injection 150 μg of anti‐CD8 antibody (clone 2.43) at day −2, −1, and 0 before challenge. In the depletion of CD4 T cells 200 μg of anti‐CD4 antibody (clone GK.1.5) similarly injected during the same time interval. To deplete both CD4+ and CD8+ T cells vaccinated mice were treated a combination of 100ug of each depletion antibody. A normal rat IgG (200 μg per mouse) was injected in a control group. The effectiveness of the depletion was assessed at day 0 prior to the challenge through staining for T cells in the lungs and spleen.

#### Statistics

Post challenge mean Vaccinia virus lung titers and mean percentage in weight loss were compared between vaccination groups using two tailed Student's *t* test. Differences were considered significant at *p* < 0.05 after analysis using prism 6 Graph Pad Software. Similarly differences in mean total tetramer binding CD8^+^ T cells were also compared using two tailed student's Test.

## Results

### Complementary prime boost vaccination with DC targeted gag protein vaccine and rNDV‐L‐gag enhances protective immunity to airway challenge with vaccinia gag

**Figure 1:**
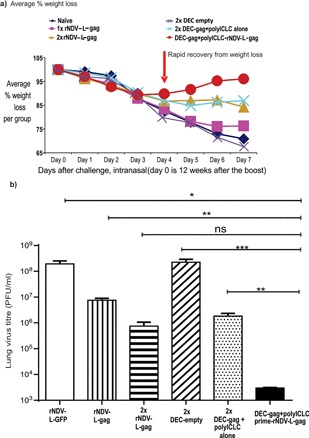
Complementary protein prime‐rNDV‐L‐gag boost vaccination results to a vigorous protection in the airway. CxB6 F1 mice were vaccinated as indicated in (a) and the x‐axis of (b) then challenged 12 weeks after the last boost. In a weight loss was monitored daily for 7 days and lung virus titer determined as previously reported 22, 23, 25, 26. (b) Mean ± SD lung virus titers (PFU/ml) of three repeat experiments are shown (*p = 0.02, **p = 0.04, ***p = 0.05, *p* = 0.08 ns, notsignificant (two tailed *t* test).

We previously demonstrated that a complementary prime boost with DC‐targeted HIV‐1 gag protein vaccine and DNA enhances protective CD4^+^/CD8^+^ T cell immunity to airway challenge with a high dose of recombinant vaccinia gag virus 22. Here we extend this concept to include the enhancement of protection afforded by combining a DC‐targeted gag protein vaccine prime and rNDV‐L‐gag boost. To test the protection afforded by protein and rNDV‐L‐gag vaccines, we first compared two doses of DC‐targeted, DEC‐gag protein vaccine to two doses of rNDV‐L‐gag vaccine, given 1 month apart in a homologous prime boost approach, and challenged 12 weeks later with a lethal intranasal dose of recombinant vaccinia‐gag virus. Weight loss was monitored daily and at days 6–7, the mice were euthanized and lung virus titers determined as previously described 22, 23, 24, 25, 27. Both protein and rNDV‐L‐gag forms of vaccine in a homologous prime boost vaccination resulted to 2–2.5 log fold less lung virus than the naïve mice (Fig. 1b).

Next, to try to improve protective immunity, we immunized mice sequentially with a single dose of DEC‐targeted gag protein vaccine followed by an intranasal boost with rNDV‐L‐gag 4 weeks later. Twelve weeks after boosting, mice were challenged with a recombinant vaccinia gag, where upon weight loss was monitored daily and lung virus titers determined as described in the Section Vaccinia‐gag protection assay. All mice lost weight during the first three days post challenge. However mice receiving either DEC‐empty or rNDV‐L‐gfp (control vaccines without gag) showed continuous weight loss. A single dose of rNDV‐L‐gag vaccine did not protect against weight loss (Fig. 1a). Mice receiving two doses of either DEC‐gag or rNDV‐L‐gag exhibited some protection against weight loss. However, priming with DEC‐gag plus polyICLC protein vaccine followed by a rNDV‐L‐gag boost provided superior protection against weight loss to either two rNDV‐L‐gag or DEC‐gag vaccines (Fig. 1a) and reduced lung virus titers by an average of 5 logs in 4 experiments (Fig. 1b), which titers were significantly lower than mice receiving a homologous prime boost vaccine (*p* < 0.02). The control for the challenge experiment were three viruses including wild type vaccinia virus with no insert (Vaccinia WT), recombinant vaccinia‐luciferase and recombinant Vaccinia‐OVA (Figure S1).

### CD4^+^ and CD8^+^ T cells protect mice after DEC‐gag protein‐prime rNDV‐L‐gag‐boost vaccine

**Figure 2:**
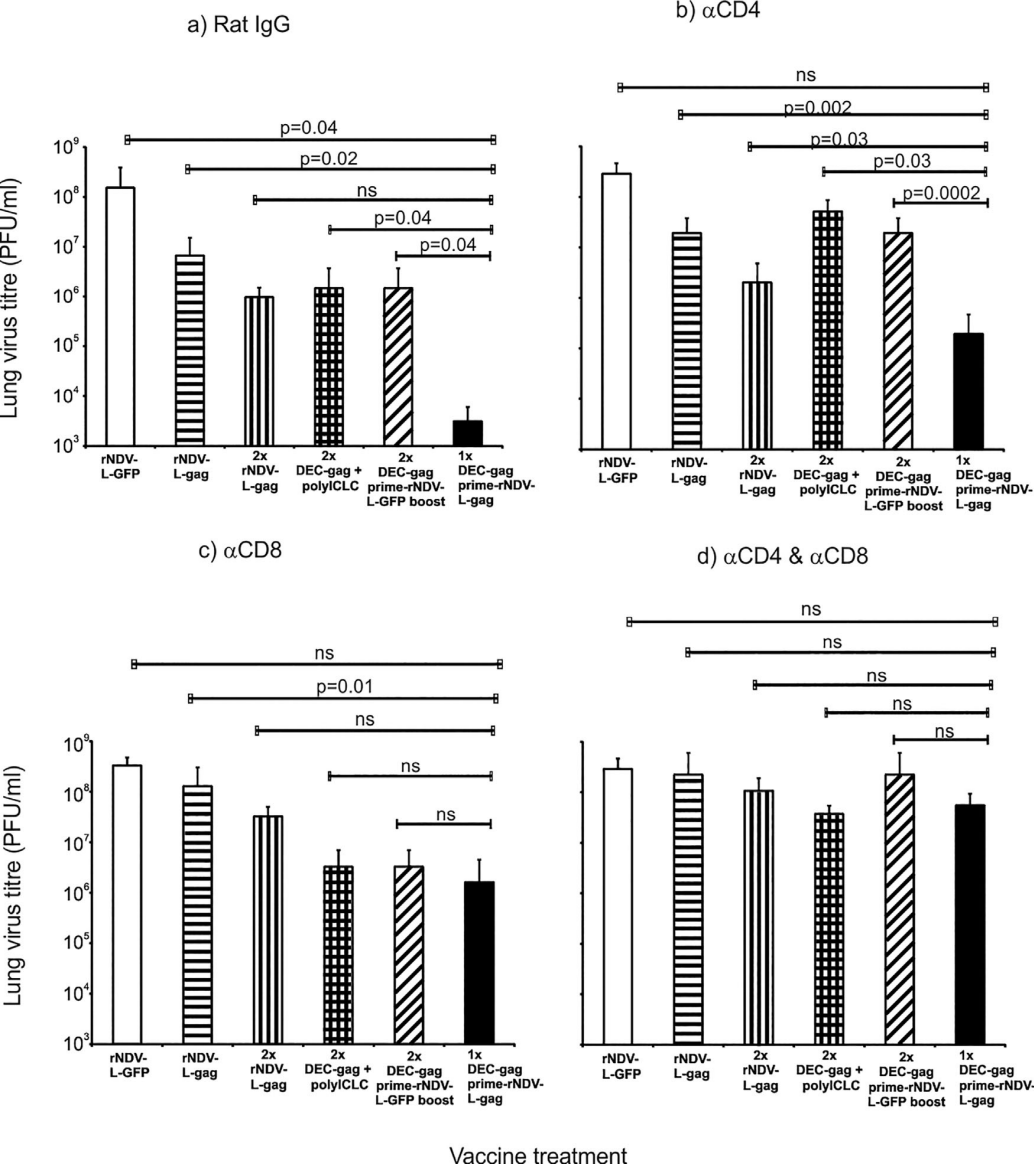
CD4^+^ and CD8^+^ T cells protect mice after DEC‐gag protein‐prime rNDV‐L‐gag‐boost vaccine. Mice vaccinated as indicated in x‐axis of Figure 3(a–d) above were treated as indicated with control rat IgG or depleting antibodies to αCD4, αCD8, or both (αCD4 & αCD8) at days −3, −2, −1 prior to airway challenge with recombinant vaccinia‐gag virus. Lung virus titer (PFU/lung) was determined 7 days after challenge with recombinant vaccinia‐gag virus (Mean ± SD of three repeat experiments) (ns, not significant (two tailed student's *t* test).

Female CxB6 F1 Mice vaccinated as indicated in the x‐axis of Figure 2 were treated either with control rat IgG (a) or depleting antibodies to αCD4, αCD8, or both (αCD4&αCD8) at days −3, −2, −1 prior to airway challenge with recombinant vaccinia‐gag virus. Lung virus titer (PFU/lung) was determined 7 days after as previously described 21, 22, 23, 24, 25, 26.

Generally a depletion of both CD4^+^ and CD8^+^ T cells abrogated protection completely in all vaccine treated groups (Fig. 2d). In Figure 2b and c the depletion of CD8^+^ T cells after homologous rNDV‐L‐gag vaccination resulted to a stronger reduction in protection, that is, a significant increase (*p* < 0.05) in lung virus titer (at least 20 fold) compared to CD4^+^ T cell depletion (threefold increase). On the other hand as shown in Figure 2b CD4^+^ T cell depletion in contrast to CD8 resulted to a 34 fold increase in lung virus titer. This indicates that protection arising from rNDV‐L‐gag vaccination is dominated by CD8^+^ T cells in contrast to DEC‐gag vaccination which is dominated as previously reported in our group by CD4^+^ T cells. Depletion of CD8^+^ T cells following complementary prime boost vaccination resulted to a 500 fold increase in lung virus titer post challenge as against 62 fold increase following CD4^+^ T cell depletion. This implies that superior protective CD8^+^ T cells are mobilized after complementary prime boost (CPB) and play a critical role in controlling the virus in the air way.

### CPB with DC targeted gag protein vaccine and rNDV‐L‐gag results to rapid and durable gag reactive CD8^+^ T cells in murine airway

**Figure 3:**
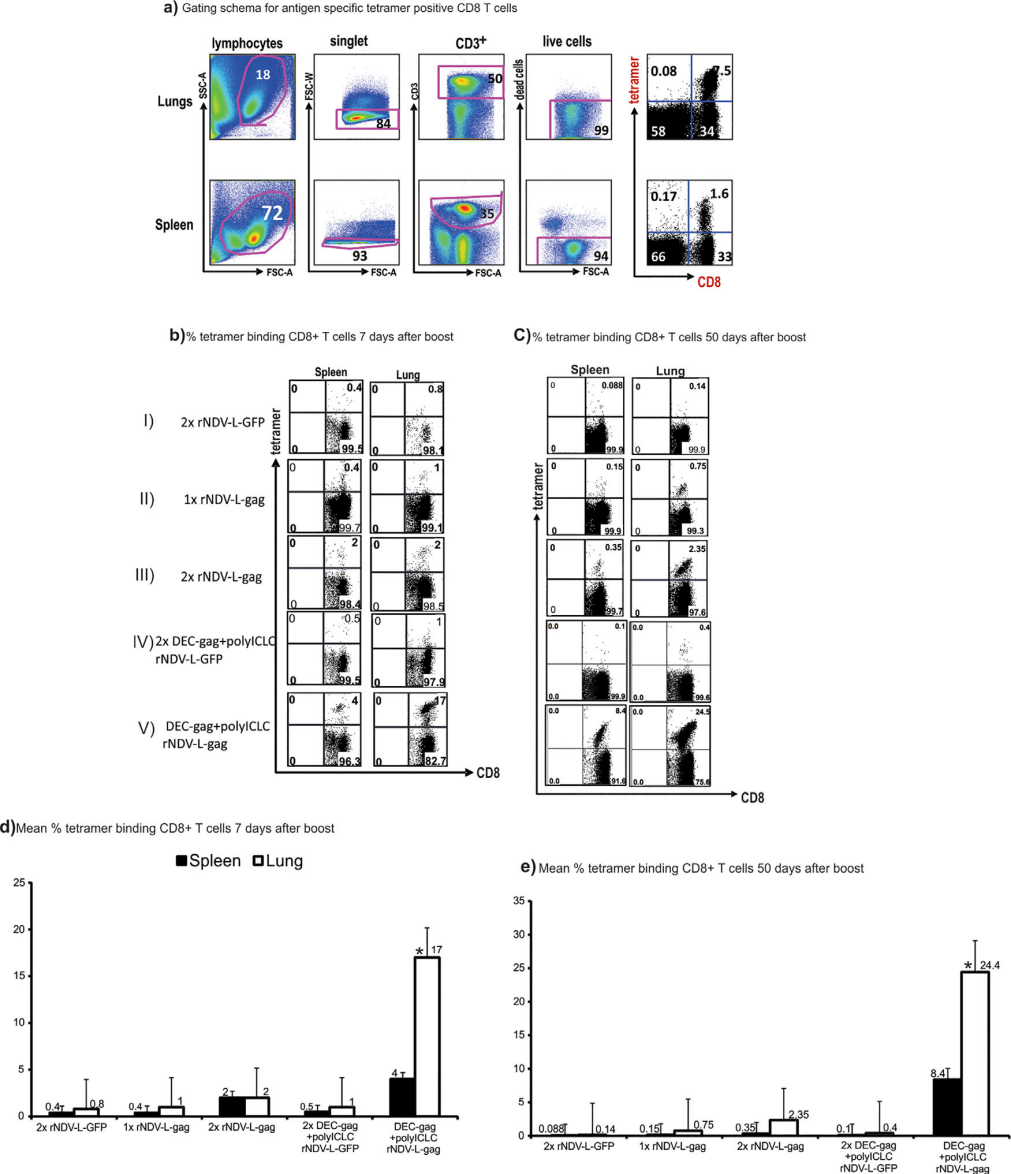
Complementary DEC‐gag protein prime rNDV‐L‐gag boost vaccine allows for enhanced accumulation of CD8^+^ T cells to an infection challenge site. CxB6F1 mice vaccinated as described in Figures 3, 4 were assessed for tetramer binding CD8^+^ T cells in the spleen and the lungs as the infection site. HIV gag specific CD8^+^ T cells were enumerated by binding of tetramers formed from H‐2Kd and HIV gag peptide (AMQMLKETI). (a) FACS data to illustrate gating schema for tetramer binding CD8^+^ T cells in lungs and spleen 10d after rNDV‐Lgag boost vaccine. (b) As in (a) Female CXB6 F1 mice were vaccinated as indicated in the y‐axis and 10 days lungs are dissociated to determine tetramer binding cells. (c) as in (a) but mean ± SD of three experiments. (d) as in (b) but 50 days after the rNDV‐L‐gag boost (*p = 0.05 student's *t* test). (e) as in (c) mean ± SD of three experiments 50 days after rNDV‐L‐gag boost.

We previously demonstrated that pre‐programming of helper CD4^+^ T cells by dendritic cell targeted HIV gag protein plus polyICLC (DEC‐gag) vaccination helped DNA vaccines improve the mobilization of protective CD8^+^ T cells in murine airway 22. Since rNDV‐L‐gag is administered via the intranasal route we tested whether DEC‐gag vaccine can also improve rNDV‐L‐gag induced CD8^+^ T cell response in murine airway. Briefly female 6–8 weeks old CXB6 F1 mice were vaccinated twice at 4 weeks apart as indicated in Figure 3b. We then asked whether the priming with DEC‐gag vaccine improved CD8^+^ T cell responses by examining the rapidity with which gag‐specific CD8^+^ T cells were mobilized quantitatively to a site of infection. In four experiments, 30 days after DEC‐gag vaccination, we boosted the mice with rNDV‐L‐gag i.n. and then examined the site of infection, the lungs, as well as the spleen 7 and 50 days later. Priming with DEC‐gagP24 plus polyICLC followed by boosting with one dose of rNDV‐L‐gag led to a rapid and better accumulation of CD8^+^ T cells to the lung (*p* = 0.05) within 7 days than a single dose or 2 doses of rNDV‐L‐gag vaccine (compares Fig. 3b rows II, III, &V).

Seven days after DEC‐gag prime followed by rNDV‐L‐gag boost CD8^+^ T cell immunity in the lungs increased 8.5 fold relative to 2x rNDV‐L‐gag vaccination. When monitored over time the CD8^+^ T cell responses persisted for well over 50 days increasing over time in both the spleen and lungs (Fig. 3d and e). When compared with the spleen CD8^+^ T cell accumulation in the lungs was at least three fold higher than the spleen after complementary prime boost vaccination (compare Fig. 3d and e).

Homologous vaccination with 2x DEC‐gagP24 plus polyICLC produced no gag specific CD8^+^ T cell responses as previously reported 23. To establish that the accumulation of gag‐reactive CD8^+^ T cells in the lungs and spleen was specific to the vaccine antigen we next vaccinated mice twice with DEC‐gag protein plus polyICLC then boosted with NDV‐L‐GFP. In the absence of gag within the rNDV vector no gag specific tetramer binding CD8^+^ T cells were detected clearly indicating that GFP as an irrelevant antigen has no effect in mobilizing HIV‐1 gag reactive CD8^+^ T cell. This is also a control to show that the rNDV vector on its own is not responsible for the expansion of pre‐existing antigen specific T cells. Thus complementary DEC‐gag prime‐ rNDV‐L‐gag boost enables a rapid and durable mobilization of CD8^+^ T cells in murine airway.

### DC‐targeted protein vaccination results to strong combined CD4^+^ and CD8^+^ T cell immunity to an rNDV‐L‐gag vaccine

**Figure 4:**
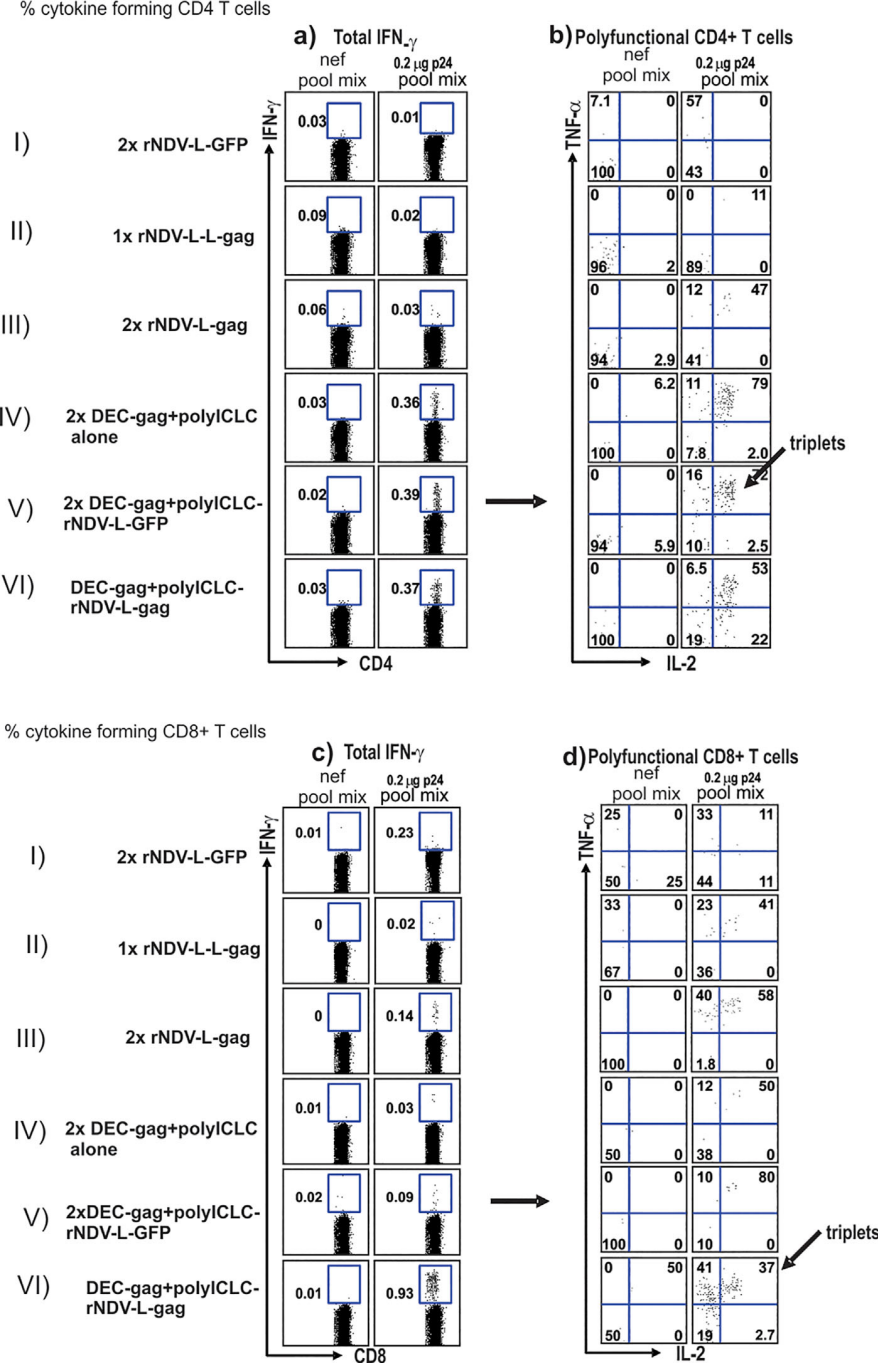
Complementary DEC‐gag protein prime rNDV‐L‐gag boost vaccination induces robust balanced CD4^+^ and CD8^+^ T cell immunity. Female CxB6 F1 mice were vaccinated as shown in the y‐axis of Figure 3, 7–10 days after the rNDV‐L‐gag boost, bulk splenocytes were assessed for T cell immunity. Splenocytes were restimulated either with unreactive peptides or with an HIV gagP24 peptide mix, and cytokine production (IFN‐γ, IL‐2, and TNF‐α) in response to peptide was evaluated by intracellular cytokine staining 6 h later in CD4^+^ (a and b) or CD8^+^ (c and d), CD3^+^ T cells. In Figure 3 total IFNγ production is shown for CD4^+^ (a) or CD8+ (c) T cells; whereas in b and c multiple cytokine forming cells are displayed for CD4^+^ and CD8^+^ T cells respectively.

To assess T cell immunity after vaccination with dendritic cell targeted gag protein followed by a rNDV‐L‐gag boost, we measured CD4^+^ and CD8^+^, gag‐specific T cells at the single cell level. One dose of rNDV‐L‐gag elicited weak CD4^+^ and CD8^+^ immunity (Fig. 4a–d, row II). In Figure 4a–d, rows III & IV data is shown for homologous vaccination with either 2x rNDV‐L‐gag or 2x DC targeted protein plus polyICLC. Whereas vaccination with 2x DEC‐gag protein plus polyICLC resulted to strong CD4^+^ T cells most of which were also simultaneously producing IFNγ, IL‐2 and TNF‐α as previously reported by our group 22, 23, 24, 25, immunization with 2x NDV‐L‐gag elicited weak CD4^+^ T Cells (compare Fig. 4a and b rows III with IV & V) and some CD8^+^ T cells. When mice vaccinated twice with DC targeted protein plus polyICLC are boosted with NDV‐L‐GFP only CD4^+^ T cell specific to the gag protein are detected clearly indicating that GFP as an irrelevant antigen would not boost preprogramed gag specific T cells. This is also a control to show that the NDV vector on its own is not responsible for the expansion of pre‐existing antigen specific T cells. On the other hand prior vaccination with DC targeted protein resulted in strong combined CD4^+^ and CD8+ T cells immunity to a single dose of NDV‐L‐gag (Fig. 4a–d, compare rows VI). Thus complementary prime boost enables a balanced induction of both CD4^+^ and CD8^+^ T cells (Figure S2).

### CPB with DC targeted gag protein vaccine and rNDV‐L‐gag elicits durable polyfunctional T cells

**Figure 5:**
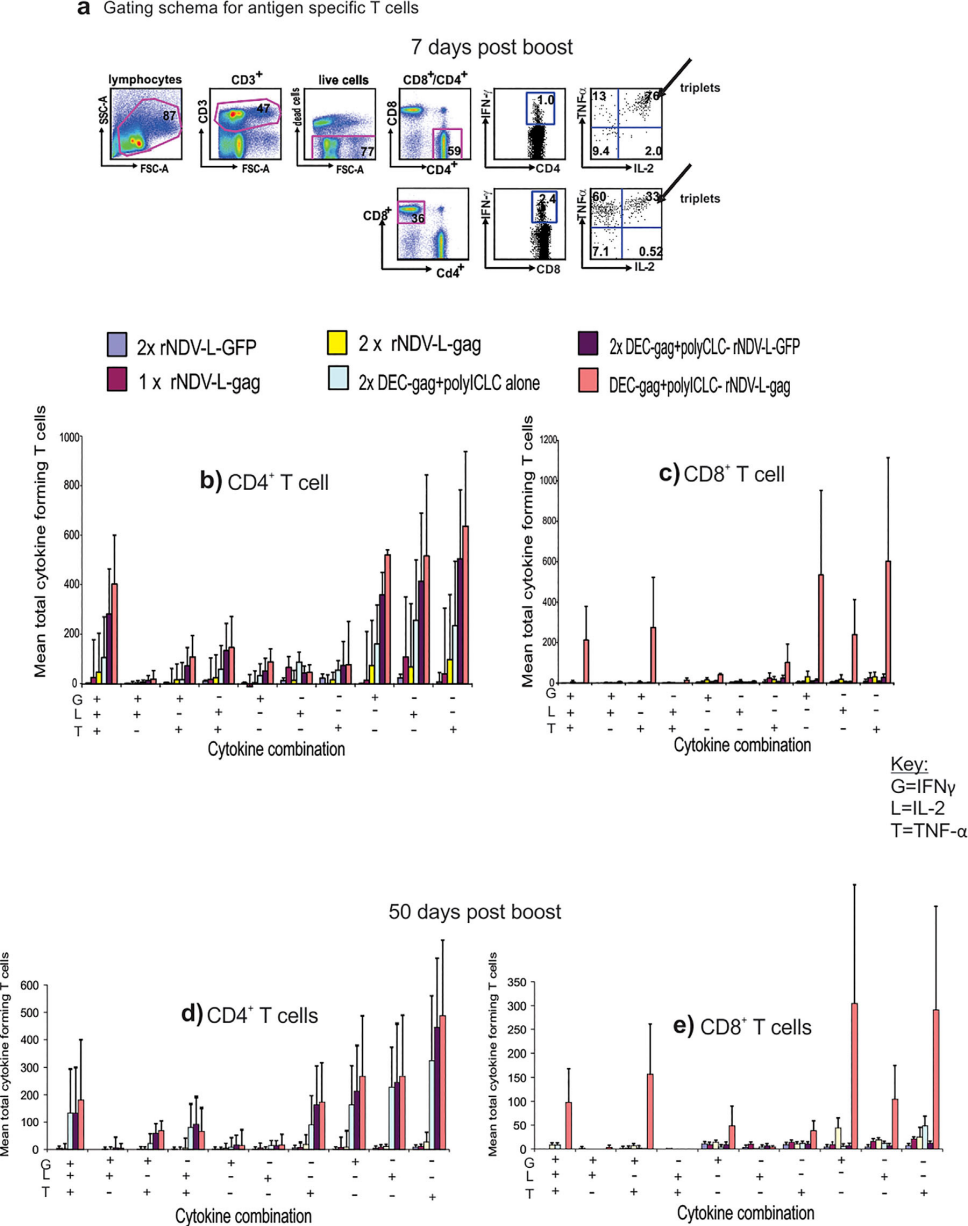
Complementary DEC‐gag protein prime rNDV‐L‐gag boost vaccination induces robust and durable polyfunctional CD4^+^and CD8^+^ T cell immunity. Female CXB6 F1 mice were vaccinated and T cell immunity measured as described in Figure 4. Cytokine production profile of single cells is assessed as shown in the gating schema in Figure 4a. Seven days after the rNDV‐L‐gag vaccine boost CD4^+^ (b) and CD8^+^ (c) T cells produce multiple cytokines including various combinations of IFN‐γ, IL‐2, and TNF‐α. Fifty days after the rNDV‐L‐gag vaccine boost, we again measured HIV‐1 gag specific cytokine production combinations in single CD4^+^ (d) and CD8^+^ (e) T cells, All data are mean ± SD of 3 experiments involving 5 F1 mice per group.

T cells producing multiple cytokines appear to be especially effective in protective immunity 9, 28, 29, 30, 31 and have been associated with long term control of HIV‐1 in elite controllers. We therefore examined the quality of the T cell responses induced by complementary DC targeted protein and rNDV‐L‐gag vaccination in terms of the ability of the gag specific T cells to secrete multiple cytokines such as IFNγ, TNF‐α, and IL‐2 (polyfunctional T cells) . Briefly female CxB6 F1 mice were vaccinated as described above and the gag specific T cells were assessed at the single cell level for their ability to secrete multiple cytokines such as IFNγ, TNF‐α, and IL‐2 (Fig. 5). In four separate experiments seven days after immunization complementary DC targeted gag and rNDV‐L‐gag vaccination enabled the induction of triple cytokine secreting cells (including IFNγ, IL‐2, and TNF‐α) by both CD4 and CD8 T cells (Fig. 5a and b). Particularly in the CD8^+^ T cell compartment cytokine formation after CPB vaccination was significantly higher (*p* < 0.003) than immunization with 2x NDV‐L‐gag.

As previously reported homologous vaccination with 2x DEC‐gag plus polyICLC vaccination resulted to strong polyfunctional T cell responses only in the CD4^+^ compartment with no detectable gag specific polyfunctional CD8^+^ T cells 22, 23, 25. As shown in Figure 5a more double cytokine forming cells were also detected in gag specific CD4^+^ T cells than in the CD8 compartment. When monitored 50d later multiple cytokine forming cells persisted in both the CD4^+^ and CD8^+^ compartment (Fig. 5c and d). Thus CPB provides improved systemic CD4^+^ and CD8^+^ T cell immunity to one dose of rNDV‐L‐gag vaccine.

## Discussion

In this study, we demonstrate that successive administration of DEC‐gag protein and rNDV‐L‐gag vaccines results to vigorous systemic and mucosal T cell immunity. Here antigen specific helper CD4^+^ T cells are elicited by a priming dose of a DEC‐targeted protein vaccine which then improves the induction of T cell immunity in murine airway by an rNDV‐L‐gag vaccine. In addition to an enhanced T cell immunity; this approach strongly amplified persistent polyfunctional T cells in the vaccinees and improved protection against airway challenge with a recombinant vaccinia gag virus. To the best of our knowledge, this is the first viral vectored vaccine strategy in mice where by the concept of helper CD4^+^ T cells has been exploited not only to direct the induction of a balanced HIV‐1 gag specific T cell immunity but equally to intentionally focus broadly reactive T cell responses at the site of pathogenic virus challenge.

This approach as previously reported in our group for DNA vaccines 22 is termed complementary prime boost vaccination because two vaccines inducing different types of immunity are combined together to achieve effective immunization. This concept is now extended here to include directing mucosal viral vectored vaccine with helper CD4^+^ T cells to focus T cell immunity at pathogenic virus entry points. However unlike our previous report where initially the was lesser vaccine specific T cells in the lungs *vis a vis* spleen 22, 32 following complementary DEC‐gag prime‐rNDV‐L‐gag vaccine boost at least 3 to 8 fold more long lasting tetramer binding CD8^+^ T cells are induced in the lungs than the spleen. In the context of HIV‐1 infection robust multifunctional mucosal T cells would be vital not only in abrogating infection but equally in limiting HIV‐1 viral reservoirs establishment through active killing of virus producing cells 7, 8, 33, 34, 35. Multiple vaccine combination strategies have been exploited to augment viral vectored vaccines mediated T cell immunity (reviewed in Ref. 36, 37, 38, 39, 40, 41), including heterologous vector prime protein boost or vice versa 42, 43, 44.

However in CPB we emphasize combining dendritic cell targeted antigen vaccination with other conventional approaches because it intensifies and broadens antigen specific immune responses 22, 23, 45, 46, 47, 48, 49, 50. Here coordinated cellular interplay between DC, CD4, CD8, B, and probably other immune cells is critical not only in shaping the quantity or quality of the vaccine mediated immune responses but also in the ultimate protection from pathogenic viral challenge. Synthetic double‐stranded RNA, polyICLC, for example, is necessary to activate DC loaded with HIV‐1 gag to induce broadly reactive helper CD4 ^+^ T cells as previously reported in our group 22, 23, 25, 49, 51. During this process IFNAR signaling in DC after polyICLC stimulation has recently been demonstrated 51 to be a pre‐requisite for the effective induction of CD4^+^ and CD8^+^ T cells. In addition evidence also indicates that this signaling activates all path ways necessary for immune induction and also reprograms DC metabolism for effective immune induction 50, 52. On the other hand the contribution of CD4^+^ T cells to the CPB strategy are numerous including our previous report 22 and several other findings demonstrating the usefulness of helper CD4^+^ T cells in T cell immunity 22, 32, 43 (reviewed in Ref. 53, 54).

The complementary prime boost approach described here, in which DC‐targeted protein vaccine favors helper cell formation and rNDV‐L‐gag vaccine favors mucosal T cell immunity limits repeated vector administration thereby reducing anti‐vector immunity as well as competition between peptides presented from vector and vaccine antigens. By directing the helper response to defined proteins, it may be feasible to amplify and improve the quality of the immunity induced by viral vectored vaccines, and augment resistance to infectious and malignant diseases. Nevertheless there is a need to assess the efficacy of this strategy in nonhuman primate models and subsequently in first in clinical trials.

## Authors' Contributions

G.N., KU, and A.W.B. designed research; G.W.N, C.G.P,L.N., and A.W.B performed research; G.W.N., G.A., L.N., N.N.N, A.A.N,A.S.O,L.A.,B.S., N.S.,G.O.C,R.U.,H.S.M, T.D.M., A.B.W., C.G.P. C.O.E., and A.S.O. analyzed data; and G.W.N., L.N., C.G.P., and A.W.B. wrote the paper.

## Conflicts of Interest

None.

## Supporting information

Additional supporting information may be found in the online version of this article at the publisher's web‐site.

**Figure S1**. Protection following complementary DEC‐gag protein prime‐rNDV‐L‐gag boost is HIV Gag dependent.

**Figure S2**. Complementary protein prime‐rNDV‐L gag boost vaccination induces equitable CD4 and CD8 T cell immunity.
